# Analysis of Pharmaceutical Active Compounds in Complex Water Samples: Sample Filtration as an Option

**DOI:** 10.3390/molecules30071609

**Published:** 2025-04-03

**Authors:** Sofia Silva, João Rodrigues, Vitor V. Cardoso, Rui N. Carneiro, Cristina M. M. Almeida

**Affiliations:** 1 Direção de Laboratórios, Empresa Portuguesa das Águas Livres, S.A.–EPAL, 1800-031 Lisboa, Portugal; sofia.silva-e@adp.pt (S.S.); vitor.cardoso@adp.pt (V.V.C.); rui.carneiro@adp.pt (R.N.C.); 2 Department of Chemistry, CICECO–Aveiro Institute of Materials, University of Aveiro, Campus Universitário de Santiago, 3810-193 Aveiro, Portugal; joao.rodrigues@ua.pt; 3 iMed.UL, Faculdade de Farmácia, Universidade de Lisboa, 1649-003 Lisboa, Portugal

**Keywords:** wastewater, PhACs, filtration, pharmaceutical compounds, pre-treatment, UPLC-MS/MS

## Abstract

Sample pretreatment is one of the most important steps in guaranteeing the success of a chromatographic analysis. The selected methodology must ensure simultaneously that a sample is “clean” enough for analysis and that the target analytes are not removed in the process. This can be especially difficult when working with complex matrices such as natural waters and wastewater. For pharmaceutical active compounds (PhACs) analysis by solid-phase extraction (SPE) followed by ultra-high performance liquid chromatography coupled to tandem mass spectrometry (UPLC-MS/MS), and due to the high level of organic matter in wastewater, the water samples are filtered consecutively through three filters, a paper filter, a glass microfiber filter of 1 µm, and a Nylon filter of 0.45 µm. This filtration allows the sample’s passage through the SPE cartridge to be faster, and there is no cartridge clogging, allowing for greater efficiency in the adsorption process. The big question is whether the PhACs are eliminated during filtration, since they may be adsorbed to organic matter. This work aimed to determine if the best approach for quantifying PhACs in wastewater and surface waters would be to filter them prior or to perform SPE directly. Both approaches analyzed a total of 26 PhACs. Turbidity (TUR) and permanganate index (PI) were determined, and their values were high for samples with a high organic matter content. A statistical analysis was performed to determine the best approach to treat these water samples and whether any correlation existed between PhAC concentrations, PI, and TUR. The PhAC quantification shows a positive correlation with TUR and a negative correlation with PI for most of the target PhACs. However, there are not significantly different results for filtered and not-filtered wastewater samples.

## 1. Introduction

The contamination of ecosystems by anthropogenic pollutants is a major threat and constraint to ecological relationships, jeopardizing ecosystem balances and, directly or indirectly, human health [1,2,3].

The treatment of many human and animal diseases depends on access to effective medicines [4,5]. At the same time, the pollution caused by some pharmaceutical compounds is an emerging problem with well-documented environmental risks, particularly concerning antimicrobial resistance to human health [6]. Pharmaceutical residues, such as pharmaceutical active compounds (PhACs), can enter the environment during manufacture, use, and disposal [2].

Therefore, the PhACs and their metabolites and degradation by-products have been detected in surface and groundwater, soil, and animal tissues throughout the European Union at varying concentrations, depending on the physic-chemical properties of the PhAC and the proximity of the contamination sources [7,8]. Certain analgesics, antimicrobials, antidepressants, contraceptives, and antiparasitic PhACs are frequently detected [9,10,11]. Traces of some PhACs have also been found in drinking water [12,13].

The impact of their presence on different environmental compartments, particularly water, is poorly understood, and there are insufficient data on their biodegradation, toxicity, and environmental fate [7].

This problem also inevitably arises with the use of pharmaceuticals by humans, whose excretion results in the release of molecules into urban wastewater which wastewater treatment plants (WWTPs) are not designed to remove, with the result that PhACs and their residues can reach the aquatic environment, where they can persist indefinitely [9]. The presence of these residues in water intended for the production of drinking water means that these molecules are ingested, even after treatment, and this issue is of particular concern in the case of groundwater [14]. The use of pharmaceuticals in veterinary practice also contributes to this dispersion in the environment, making the study of their potential effects a challenge for the scientific, academic, and industrial communities [15].

Extreme variations in raw water availability and quality, as well as the deterioration of water sources, are being caused by this problem in combination with other human-induced stresses and climate change. From a technological, political, and social perspective, these issues are a challenge for the relevant authorities. These challenges mostly relate to monitoring the presence of these substances, evaluating their potential hazardous consequences, and, most importantly, finding ways to eliminate or reduce any potential negative impacts. The most common problems related to their potential harmful effects are mutagenicity, endocrine disruption, and antibiotic resistance [14].

In addition to reducing this waste for more environmentally sustainable use, there is an urgent need to characterize the presence of pharmaceutical residues and quantify safety levels, forms of absorption, and potential toxicological effects. Therefore, high-quality monitoring data and data on ecotoxicological and toxicological effects are needed for risk assessments to support the selection of new priority substances. Although significantly improved in recent years, the quality and Union coverage of the monitoring data collected by the Member States is not always adequate. Monitoring data are particularly lacking for many emerging pollutants, which can be defined as pollutants that are not currently included in routine monitoring programs at the Union level. However, they may be a significant risk in terms of their potential ecotoxicological risk and therefore in need of regulation [11,16].

In water policy, Directive 2013/39/EU for the first time identifies the contamination of water and soil by pharmaceutical waste as an environmental problem [17]. In order to ensure the monitoring of substances in Europe that may pose a risk, such as emerging pollutants, and to ensure a high-quality database for the identification and prioritization of these substances, this Directive establishes a list of priority substances that will be updated on an ongoing basis. The watch list system was created to require temporary oversight of various substances for which data indicated a potential environmental risk, in order to assist in the selection of further priority substances. The watch list must contain no more than 10 substances or groups of substances, specify the matrices to be monitored, and propose cost-effective analytical methods. Concerning PhACs, this first watch list includes a non-steroidal anti-inflammatory drug (diclofenac) and the hormones 17-β-estradiol (E2) and 17-α-ethinyl estradiol (EE2), to assess concentration levels at which appropriate measures can be taken in view of the risks posed by these compounds.

The first watch list was established by Decision 2015/495/EU of 20 March 2015 [18] and consists of seven PhACs: two natural hormones (E2 and estrone (E1)), one synthetic hormone (EE2), one nonsteroidal anti-inflammatory drug (NSAID) (diclofenac), and three macrolide antibiotics (erythromycin, clarithromycin, and azithromycin).

This Directive was repealed by Decision 2018/840/EU (30) of 5 June 2018, and the substances listed above (EE2, E2, E1, azithromycin, clarithromycin, and erythromycin) were included together with two additional antibiotics, amoxicillin and ciprofloxacin. The Commission removed diclofenac from the watch list because of the availability of good quality monitoring information on diclofenac [19]. The addition of amoxicillin and ciprofloxacin is in line with the European One Health Action Plan Addressing Antimicrobial Resistance (AMR), which advocates for the use of the watch list to ‘enhance understanding of the presence and distribution of antimicrobials in the environment’ [20].

The last watch list was established by Decision 2022/1307/EU of 22 July 2022, and listed nine PhACs and some metabolites, namely sulfamethoxazole, trimethoprim, venlafaxine and O-desmethylvenlafaxine, clotrimazole, fluconazole, miconazole, clindamycin, ofloxacin, metformin, and guanylurea [21].

All assessments and decisions were supported by occurrence studies of these pollutants in the aquatic environment, where high-quality information on their levels (quality of the analytical results) was critical. Moreover, understanding their concentration is essential as a basis for implementing more sophisticated treatments to enhance their elimination and, as a result, reduce their environmental threat [22].

In this context, analytical chemistry is essential to provide high-quality data through two distinct approaches: (i) target analysis and (ii) screening techniques for identifying non-target or unknown substances (degradation products). Both methods require advanced techniques to apply sensitive and selective processes to provide precise information on the identification and quantification of compounds [11,23].

In this setting, analytical challenges, particularly in quantitative analysis, focus on the development and validation of new materials, approaches, and procedures to swiftly fulfil the demands for selectivity, sensitivity, speed, and environmentally friendly methods. Due to the ongoing updates of international standards, guidelines, and recommendations, continuous innovation in pre-treatment and instrumental settings is essential to achieve dependable, accurate, and reproducible data [24].

Regardless of the chromatographic method used, sample pre-treatment is a crucial and often challenging step, especially for complex matrices such as wastewater. Although solid-phase extraction (SPE) is the most widely used technique for sample cleanup and analyte concentration, this step is usually preceded by sample filtration.

In natural waters (surface) and wastewater, filtration is carried out using filters compatible with the sample but with different porosities, namely glass fibre membrane filters with a pore size of 1.0 μm and nylon or PTFE (polytetrafluoroethylene) membrane filters with a pore size of 0.45 µm. This filtration prevents clogging of the SPE cartridges, thereby increasing the efficiency of the extraction process [25].

The problem is whether filtration will eliminate some target compounds because they are adsorbed to the particulate organic matter retained in the filtration process.

The answer to this question lies in evaluating the efficiency of the extraction process with and without pre-filtered samples before the SPE procedure, followed by the use of the chromatographic method to quantify the target compounds.

Therefore, this study addressed three main objectives: (i) to monitor the occurrence of 26 PhACs in the influent and effluent of several Portuguese WWTPs; (ii) to monitor the same 26 target PhACs in upstream and downstream surface waters; and (iii) to compare the profile of target PhACs in both water samples (surface and wastewater) with and without a filtration step before the SPE procedure. The target PhACs belong to ten target therapeutic classes, namely non-steroidal anti-inflammatory drugs (NSAIDs) such as diclofenac—DCF, ibuprofen—IBUP, and naproxen—NPX, analgesic drugs (acetaminophen—APAP), beta-blockers (atenolol—ATN, metoprolol—MTPL, and propranolol—PPNL), anti-dyslipidemic drugs (clofibric acid—CFA and bezafibrate—BZF), sexual hormones (17-alpha-ethynilestradiol—EE2, beta-estradiol—E2, estrone—E1, estriol—E3, diethylstilbestrol—DES, gestoden—GTD, and testosterone—TTE), antidepressant drugs (fluoxetine—FLX), anticonvulsant drugs (carbamazepine—CBZ), psychostimulant drugs (caffeine—CAF), corticosteroid drugs (cortisone—CTS), and antibiotics (erythromycin—ERT, sulfadiazine—SDZ, sulfamethoxazole—SMX, sulfapyridine—SPD, clarithromycin– CLR, and azithromycin—AZM).To increase the sensitivity of the analysis, two chromatographic methods were employed: one using a basic solvent to quantify 12 of the 26 PhACs (basic chromatographic method) and a second using an acidic solvent to quantify the remaining 14 compounds (acidic chromatographic method) [8,9].

This research was carried out at EPAL (Empresa Portuguesa das Águas Livres, SA), the leading national company in the water supply sector and a key component of Grupo Águas de Portugal (AdP). This water supply company has a significant impact on the environmental sector in Portugal and is involved in water supply and wastewater sanitation.

## 2. Results and Discussion

### 2.1. PhACs Profile in Wastewater and Surface Water

This section presents the concentrations, percentage of positive samples, and frequency of detection of PhACs in all target samples. The results encompass the minimum (Min) and maximum (Max) concentrations, the median concentration (Med), the percentage of positive samples (%Pos), and the detection frequency (Freq; the number of positive samples divided by the total number of WWTPs investigated). All PhACs not detected in the water samples, i.e., with concentrations below the method detection limit (<MDL) were excluded from the statistical analysis. The results obtained for the influent and effluent of each WWTP and the corresponding upstream and downstream surface waters are presented in Tables S1 and S2 and Tables S3 and S4, respectively.

Regarding the occurrence of PhACs in the influents of the target WWTPs, 20 out of the 26 PhACs analyzed were quantified in filtered and unfiltered samples (Table S1). Five PhACs (CFA, E1, E2, EE2, and DES) were not quantified in either of the wastewaters. GTD was only quantified in filtered samples and FLX in unfiltered samples.

The most representative PhACs were the analgesic drug acetaminophen (APAP), the psychostimulant caffeine, and the NSAIDs ibuprofen, naproxen, and diclofenac, with median concentrations in filtered samples of 61.9 µg/L, 35.7 µg/L, 8.8 µg/L, 4.45 µg/L, and 1.85 µg/L, respectively (Table S1).

The most representative PhACs in the unfiltered influent samples were also the APAP, caffeine, and the NSAIDs ibuprofen, naproxen, and diclofenac, with median concentrations of 47.9 µg/L, 35.2 µg/L, 6.5 µg/L, 3.85 µg/L, and 1.93 µg/L, respectively (Table S1).

For the remaining PhACs, the median concentration in the influent filtered samples ranged from 0.032 µg/L (testosterone) to 0.626 µg/L (sulphapyridine). The median concentration of antibiotics was greater than or equal to 0.1 µg/L, with values ranging from 0.099 µg/L (erythromycin) to 0.626 µg/L (sulphapyridine). For unfiltered samples, the median concentration ranged from 0.056 µg/L (cortisone) to 0.654 µg/L (estriol). The median concentration of antibiotics was higher than 0.1 µg/L, with values ranging from 0.152 µg/L (clarithromycin) to 0.331 µg/L (azithromycin). Erythromycin was not detected in unfiltered influent samples.

Nine of the 26 PhACs quantified in the filtered influent of WWTPs showed a high frequency of detection (about two samples by WWTP) with positive samples greater than 80% (ATN, APAP, SPD, CAF, CBZ, NPX, BZF, IBUP, DCF, and AZM). Six PhACs showed the lowest detection frequency (<15%) in these filtered samples (SDZ, CTS, GTD, TTE, E3, and ERT). The profile of positive samples was the same in unfiltered samples.

Regarding effluents (Table S2), filtered and unfiltered wastewater samples showed a quite similar profile, and eleven out of twenty-six PhACs were not quantified in these samples (SDZ, CTS, CFA, GTD, E1, E3, E2, EE2, DES, ERT, and FLX). Only five PhACs showed more than 80% positive samples (CAF, SMX, CBZ, NPX, and DCF).

Fourteen PhACs were quantified in filtered and unfiltered samples (Table S2). Twelve PhACs were not quantified in either of the wastewaters.

The most representative PhACs in the wastewater effluent were diclofenac, carbamazepine, and ibuprofen, with median concentrations of 1.680 µg/L, 0.447 µg/L, and 1.257 µg/L in filtered samples and 0.454 µg/L, 0.233 µg/L, and 0.176 µg/L in unfiltered samples, respectively. The median concentration of the remaining PhACs ranged from 0.050 µg/L (PPNL) to 0.257 µg/L (BZF) in filtered samples and from 0.039 µg/L (ATN) to 0.097 µg/L (CLR) in unfiltered samples.

Upstream surface waters showed median PhAC concentrations between 0.060 µg/L (SMX) and 11.89 µg/L (APAP) in filtered samples (Table S3). Unfiltered samples showed median PhAC concentrations between 0.085 µg/L (APAP) and 1.57 µg/L (NPX). Only ten PhACs were quantified in filtered and unfiltered samples. Most of the PhACs were not detected in these surface waters. The positive samples for the majority of the PhACs were less than 20% for both filtered and unfiltered samples.

Downstream surface waters showed median PhAC concentrations between 0.026 µg/L (ATN) and 1.01 µg/L (IBUP) in filtered samples (Table S4). Unfiltered samples showed median PhAC concentrations between 0.040 µg/L (NPX) and 1.40 µg/L (IBUP). Only nine PhACs were quantified in both filtered and unfiltered samples. Most of the PhACs were not detected in these surface waters.

### 2.2. PhACs Retention Rate

**Figure 1:**
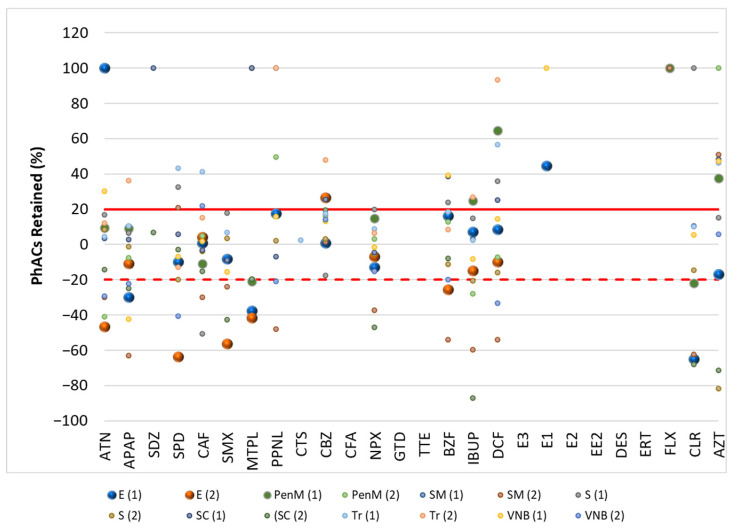
Retention rate (%) of PhACs in the filtration procedure in WWI of target WWTPs.

**Figure 2:**
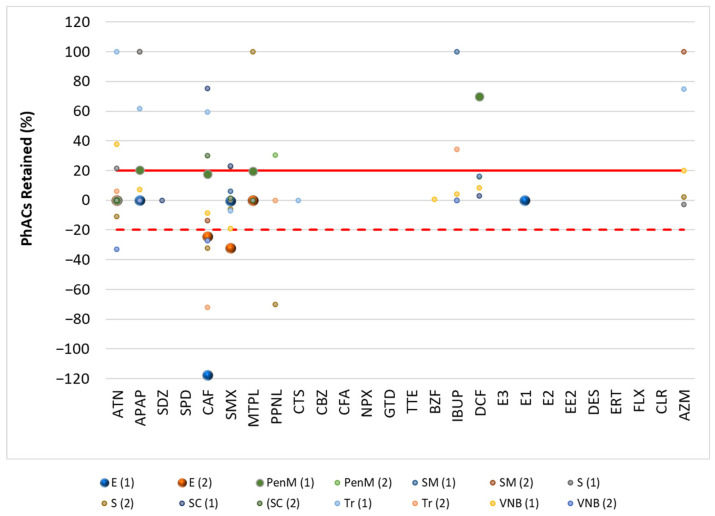
Retention rate (%) of PhACs in the filtration procedure in WWE of target WWTPs.

**Figure 3:**
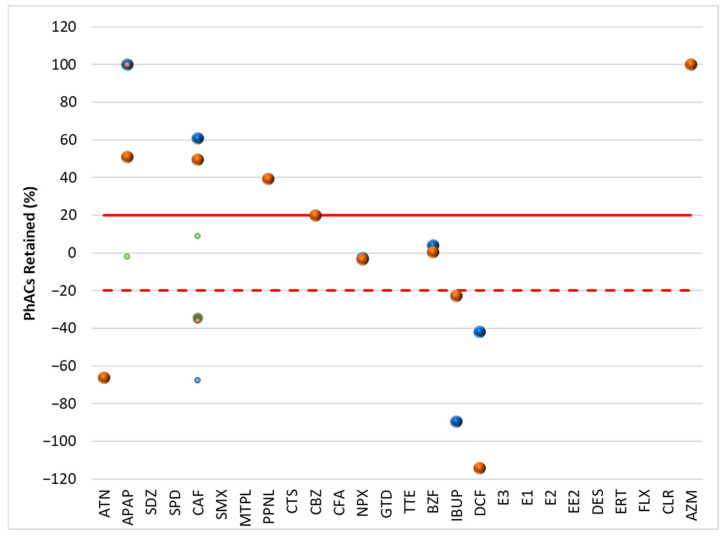
Retention rate (%) of PhACs in the filtration procedure in upstream waters of target WWTPs.

**Figure 4:**
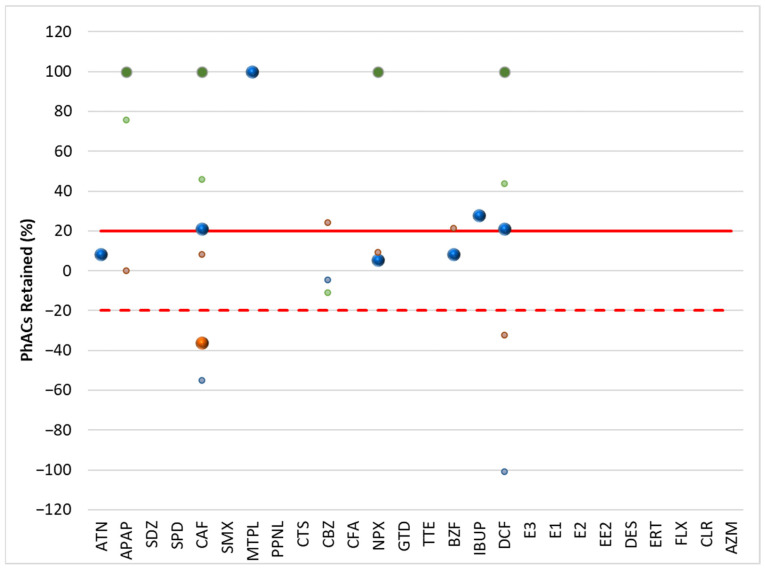
Retention rate (%) of PhACs in the filtration procedure in downstream waters of target WWTPs.

Figure 1 and Figure 2 show the results of the retention of PhACs in filtration procedures in the influent (WWI) and effluent (WWE) of the target WWTPs, respectively. Figure 3 and Figure 4 show the results of the retention of PhACs in filtration procedures in upstream and downstream waters of target WWTPs, respectively.

The filtration process resulted in various changes in the concentrations of PhACs. A positive retention percentage indicates that the PhAC’s concentration was higher in unfiltered water samples. This indicates that the compound was retained during the filtration process. Due to the high levels of organic matter in the samples, the PhAC may be adsorbed to this organic matter and retained during filtration.

A negative retention percentage indicates that the PhAC concentration was higher in the filtered water sample, so the PhAC was not adsorbed to the organic matter and it had a higher affinity for the SPE cartridge.

These results (Figure 2 and Figure 3) indicate that some PhACs have strong interactions with the filtration procedure, either through adsorption (e.g., hydrophobic interactions), size-based retention due to the interaction with organic matter, or lower affinity to the SPE cartridge (positive retention rates).

Fluctuations in retention rates of around 20% were within the range of the method’s trueness and were therefore insignificant. Therefore, only rates above 20% or below −20% were considered relevant.

In the WWE and WWI, most PhACs showed retention rates below 20% for the different compounds and WWTPs. However, there were many PhACs with retention rates above 20% and below −20% in the effluents. This behavior was independent of the PhACs and the WWTP. Although less representative (lower frequency/positives per WWTP), the WWE had a higher representation of positive retention rates.

Although surface waters (Figure 3 and Figure 4) are less representative due to the lowest number of PhACs (<frequency and positive samples), most showed retention rates above 20% in upstream and downstream waters.

Because the WWTP effluent has little effect on the downstream waters, the profile of PhAC retention rates in the filtration process of the upstream and downstream waters of the target WWTPs is quite comparable. Since the water’s quality and PhAC concentrations are comparable, the filtering process’s impact is likewise comparable.

Given the varied behavior of PhACs for different wastewaters and WWTPs, a statistical analysis was applied.

### 2.3. Turbidimetry and Permanganate Index

The complexity of the target wastewater and surface water was assessed using two water quality parameters, the turbidity and permanganate index (Table 1).

In the first sampling period, turbidity values in the WWI ranged from 40.9 NTU (VNB) to 734 NTU (SC). In the second sampling period, the values ranged from 91.4 NTU (PenM) to 500 NTU (SC).

In the first sampling period, the turbidity values in the WWE ranged from 1.36 NTU (E) to 150 NTU (Tr). In the second sampling period, the values ranged from 0.7 NTU (E) to 17.37 NTU (Tr).

The turbidity values in the WWI were of the same order of magnitude in the two sampling periods at the different WWTPs, except for the VNB WWTP. In the latter, turbidity was about 10 times higher in the second sampling period (40.9 NTU vs. 395 NTU).

In the WWE, the second sampling’s turbidity values were significantly lower. As expected, the turbidimetry values in WWI were much higher than in WWE.

The turbidimetry values in the second sampling period for upstream and downstream surface waters showed lower values for Tr and SM, indicating a decrease in these parameters, possibly due to the sedimentation of inorganic or organic material under different conditions. These values suggest that there were no significant differences between upstream and downstream surface waters. Therefore, these WWTPs did not have a significant impact on the quality of surface water.

In WWI, the values of PI between the two sampling periods did not fluctuate as dramatically as those in WWE. This suggests that WWI conditions had a more consistent level of pollution, whereas WWE had significant variations due to treatments in the WWTPs.

The upstream samples showed the highest PI values, especially for Tr (282.7 mg/L O_2_), SM (224.2 mg/L O_2_), and PenM (255.1 mg/L O_2_).

This suggests that the water was most polluted at the upstream source, indicating a significant presence of oxidizable substances whose levels were unaffected by WWE.

### 2.4. Turbidimetry and Permanganate Index Versus PhAC Quantification

**Figure 5:**
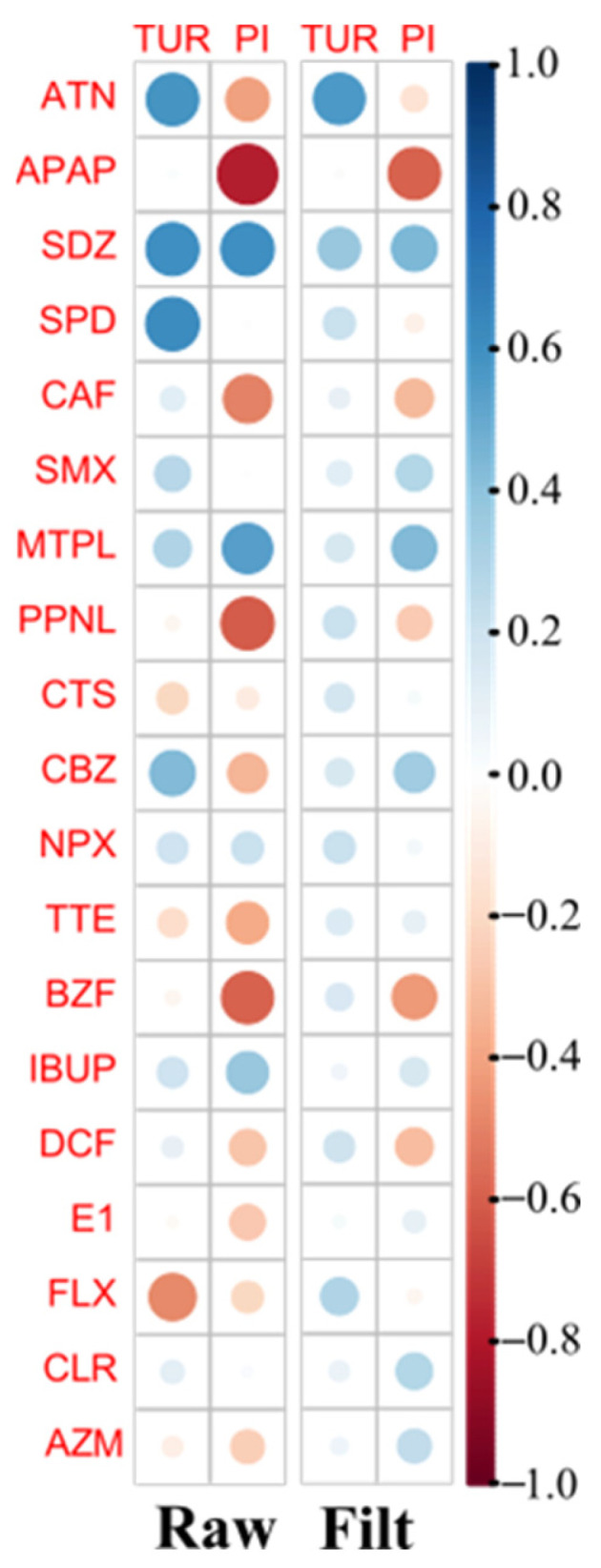
Pearson correlation plot between PhACs concentration data and specific water parameters data (turbidity, TUR, and permanganate index, PI) for raw (**left**) and filtered (Filt, **right**) WWI samples. Positive and negative correlations are shown in blue and red color, respectively.

The influence of suspended solids and organic matter on the quantification of PhACs was evaluated using Pearson correlation coefficients between the specific water parameter levels and the concentration of individual PhACs (Figure 5).

The solid-phase extraction (SPE) method is based on the adsorption of target analytes (PhACs) onto a sorbent phase, which can be influenced by the sample composition. High turbidity (suspended particles) or a high permanganate index (high organic content) can reduce SPE efficiency by (a) blocking active sites on the sorbent, (b) altering the pH or ionic strength of the solution, and (c) leading to co-extraction of unwanted substances, affecting the final analysis [11]. Therefore, high turbidity may cause physical blockage of the SPE sorbent, and a high permanganate index indicates the presence of competing organic matter that may reduce PhAC retention on the SPE cartridge.

However, Figure 5 shows a positive correlation between TUR and the concentration of most of the target PhACs and a negative correlation between PI and most of the target PhACs.

The positive correlation between PhAC concentrations and TUR suggests that suspended particles and colloidal matter in the water influence the presence, stability, or extraction of PhACs. There are several possible explanations for this correlation, namely, PhACs binding to particulate matter, the increased retention of PhACs in water, and the behavior of some PhACs during the filtration step.

Some PhACs, especially hydrophobic or poorly soluble ones, tend to adsorb on suspended particles (such as organic matter, sediment, or colloidal material) [11]. Therefore, as turbidity increases (due to more suspended solids), more PhACs can co-exist in the particulate phase, leading to higher apparent concentrations when analyzed. This is particularly relevant for more lipophilic PhACs, such as non-steroidal anti-inflammatory drugs (NSAIDs) and antibiotics, which may have an affinity for organic or clay-based particles. Organic matter can also form complexes with PhACs, slowing their degradation, photolysis, or breakdown and allowing them to persist in water. As a result, higher TUR may be associated with increased PhAC concentrations simply due to their reduced degradation [26].

For wastewater samples with high TUR, particle-bound PhACs may be co-extracted during SPE, leading to higher measured concentrations. If filtration is not performed adequately, particle-bound pharmaceuticals may be released into solution during extraction, artificially increasing their measured levels. Conversely, in clearer water samples, free-dissolved pharmaceuticals may be less efficiently retained by the SPE cartridge if competition from particulates is absent.

The negative correlation between the quantification of some PhACs and the PI suggests that oxidizable organic matter in water influences PhAC stability, degradation, or extraction efficiency. A higher PI often correlates with high levels of organic matter, humic substances, and other oxidizable organics, which compete with PhACs for adsorption sites on an SPE cartridge, reducing the efficiency of extraction and leading to lower quantification (APAP, CAF, PPNL, and BZF). The presence of oxidized organic compounds may interfere with analytical methods (e.g., LC-MS/MS), leading to an underestimation of PhAC levels.

Notwithstanding the strong negative correlation between PhAC concentrations and PI and the positive correlation with TUR, this behavior is not the same for all compounds due to the diversity of chemical structures of the 11 therapeutic classes studied. This behavior is more pronounced in unfiltered (raw) water than in filtered water due to the higher concentration of suspended solids in raw water.

### 2.5. Multivariate Analysis of WWI (Raw vs. Filtered Waters)

**Figure 6:**
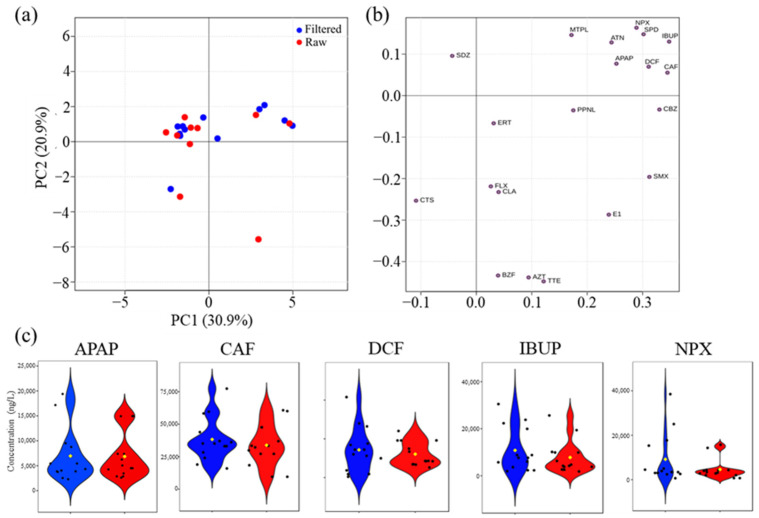
(**a**) PCA scores scatter plot obtained for PhAC concentrations in the following wastewater influents (WWI): raw (red) and filtered (blue); (**b**) PCA loading plot of PCA model shown in (**a**); and (**c**) Violin plots of the top five PhACs with highest concentration found in WWI (APAP, acetaminophen; CAF, caffeine; DCF, diclofenac; IBUP, ibuprofen; and NPX, naproxen): raw (red) and filtered (blue).

Most raw and filtered samples (Figure 6a) had very similar principal component values, meaning the filtering process did not significantly change their concentration. The PCA of the PhAC data in raw and filtered WWI (Figure 6a) revealed no clear distinction between the two profiles. This result was expected, suggesting that the variability induced by different wastewater treatment plants outweighs the influence of sample filtration on the overall WWI profile.

However, the filtering may selectively impact certain compounds while leaving others relatively unchanged, perhaps due to the different concentration of target compounds in the samples. If a raw sample already has a low level of a target PhAC, filtering might not produce a noticeable difference. However, certain PhACs with a higher concentration may be more susceptible to filtering, resulting in separation from others (Figure 6b). Nevertheless, a slight separation of a subset of WWI samples (positive PC1), primarily composed of filtered samples, was observed. The corresponding loadings plot (Figure 6b) suggests that this trend is driven by variations in certain PhACs, mainly APAP, ATN, DCF, CAF, IBUP, SPD, and NPX. Notably, five of these compounds (APAP, CAF, DCF, IBUP, and NPX) are among the top five PhACs with the highest concentrations in WWI.

Violin plots (Figure 6c) further confirm that there are no significant statistical differences between raw and filtered WWI samples present for the top five PhACs. However, filtered samples show higher concentration values of these PhACs in certain WWIs, particularly for DCF, IBUP, and NPX, suggesting that filtration may enhance the detection of these compounds under specific conditions.

## 3. Materials and Methods

### 3.1. Wastewater Treatment Plants (WWTPs), Surface Water, and Sampling Points

Seven Portuguese WWTPs were selected for this study: Torrão (Tr), São Miguel (SM), Penamacor (PenM), Seia (S), Vila Nova da Barquinha (VNB), Entroncamento (E), and Santa Cita (SC). Samples were collected from the influent, effluent, and receiving waters of each WWTP (both upstream and downstream). Four WWTPs—SM, S, PenM, and Tr—were located in the Beira Alta region, while the other three—E, SC, and VNB—were located in the Beira Baixa region of Portugal. Sampling of the influent and effluent of these WWTPs was evaluated in two sampling campaigns conducted between May and November 2023. Sampling of surface water was conducted during two campaigns, one in July and another in November.

Wastewater samples were collected as 24 h composite samples, with a 15 min sampling interval (200 mL per hour), totaling 4.8 L. For surface water sampling, punctual samples were taken instead due to the location of some sampling points.

All PhACs were quantified by SPE-UPLC-MS/MS in filtered and unfiltered water samples. The monitoring campaign included a total number of 42 samples. The selected sampling points included influents and effluents of each wastewater treatment plant (WWTP) and, when feasible, surface waters upstream and downstream of the WWTPs. Table 2 shows the characterization of the target WWTPs and the number of water samples collected.

The effect of filtration versus no filtration was studied in both wastewater and surface water, more specifically in the downstream and upstream receiving waters of each WWTP, to determine if the matrix’s complexity might affect the results.

The largest and most representative of the selected WWTPs is the Santa Cita WWTP, located in the Tomar municipality, which covers an area of 351.2 km^2^ and serves 79,832 population equivalents.

### 3.2. Wastewater and Surface Water Characterization

The evaluation of organic matter in the wastewater (influent and effluent) and surface waters (upstream and downstream) were quantified by turbidimetry and permanganate index. Turbidity was determined by nephelometry (SMEWW 2130 B) [27] using a Hach 2100Qis portable turbidimeter supplied by Hach (Carnaxide, Portugal). Turbidity levels were expressed in nephelometric turbidity units (NTUs). The equipment was calibrated and controlled with several formazin turbidimetric standards (0.02, 20, 100, and 800 NTUs) supplied by Hach (Carnaxide, Portugal).

Potassium permanganate (KMnO_4_) and potassium dichromate (K_2_Cr_2_O_7_) are two oxidizing reagents used to quantify organic compounds by two different methods, permanganate index (or oxidability) and chemical oxygen demand (COD), respectively. The permanganate index was chosen as the water quality parameter for the quantification of organic matter in wastewater and surface water. This titrimetric method [28] uses potassium permanganate 0.01 M and oxalic acid 0.01 M as titrants. Both reagents were supplied by Carlo Erba (Milan, Italy).

These determinations were performed in water samples without filtration. For WWI, the TUR and PI were determined in both filtered and unfiltered (raw) wastewater samples.

### 3.3. Water Sample Filtration

Upon reception and within 7 days, 500 mL from the samples was filtered through quantitative filter paper to remove larger particles, followed by a sequential vacuum filtration through a 1.0 μm glass-fiber filter (Type 2, Millipore, Sigma-Aldrich, St. Louis, MO, USA) and a 0.45 μm cellulose nitrate membrane (Millex 0.45 μm, Millipore, Sigma-Aldrich). Of these 500 mL, 50 mL were collected for immediate SPE. Consequently, only the concentration of PhACs in the dissolved portion of the sample was considered in the analysis when these multiple filtrations were performed. These filtration steps were performed to ensure that each sample was sufficiently clean to be extracted into SPE cartridges.

To determine whether the decision to quantify PhACs only in the dissolved fraction of the sample would have a significant impact on the obtained results, an SPE extraction of the samples was performed in parallel after filtering through quantitative filter paper to remove larger particles that would likely obstruct the SPE cartridges.

From the 4.8 L composite sample, 500 mL was filtered to ensure the sample was more representative for the SPE. However, only 50 mL of a sample was required for the SPE test.

### 3.4. SPE-UPLC-MS/MS Method

The quantification of PhACs in wastewater and surface water was performed by ultra-performance liquid chromatography–tandem mass spectrometry (UPLC-MS/MS) after the clean-up and concentration of the target compounds by solid-phase extraction (SPE), which was previously implemented and validated by our research group [9,13,29] The validated method provides linear calibration curves of all target PhACs over a concentration range from 0.76 to 87 µg/L with a determination coefficient (R^2^) between 0.9983 and 0.9999 and a coefficient of variation (CVm) between 1.6% and 6.7%. The method detection limits (MDLs) and method quantification limits (MQLs) ranged from 6.81 to 223 ng/L and from 22.5 to 736 ng/L, respectively, for water samples of different natures. Moreover, the relative standard deviations (RSDs) of the method were less than 24% for inter-day repeatability. No legislation defines acceptance criteria for the performance of analytical methods applicable to wastewater. However, legislation exists for other complex matrices such as food. According to Regulation (EU) 2021/808, the acceptance criterion for repeatability is 30% [30]. Therefore, the obtained RSDs were good. The extended uncertainty (U) of the SPE-LC-MS/MS method was less than 41%, with a 95% confidence level [31].

The PhACs in water samples were quantified by the standard addition method and their results were expressed in µg/L. Only samples with a PhAC concentration higher than the MQL were considered positive; concentrations lower than the LOQ were disregarded. Only positive samples were used for the statistical analysis.

### 3.5. Retention of PhAC Compounds During Filtration Steps

The retention rate of PhACs in filtration steps was calculated by using Equation (1).

(1) $$Retention\;rate\;(\%)=\left[\frac{\left(C_{Unfiltered}-C_{Filtered}\right)}{C_{Unfiltered}}\right]\times 100$$

where $C_{Unfiltered}$ is the concentration of PhAC in the unfiltered water samples and $C_{Filtered}$ is the concentration of PhAC in the filtered water samples.

PhACs with high retention rates indicate high removal by the filtration procedure, probably due to sorption onto sludge and low affinity to the SPE cartridge. PhACs bound to organic matter have a lower affinity for binding to the adsorbent in the SPE cartridge because their direct interaction with the adsorbent material is hindered. The organic matter may occupy the active sites of the adsorbent, reducing the available binding sites for free PhACs.

PhACs with a negative retention value indicate that the concentration of the compounds has increased, possibly due to the desorption from organic matter during the filtration step or higher affinity to the SPE cartridge than to the cartridge.

### 3.6. Statistical Analysis

Principal component analysis (PCA) [32], an unsupervised multivariate data analysis method, was applied to provide an overview of the behavior of the PhAC concentrations in wastewater influents (raw vs. filtered). The PCA was performed using MetaboAnalyst 6.0 [33], with concentration matrices of WWI PhACs scaled to unit variance (UV) to minimize the effect of different concentration ranges. The results were visualized using factorial coordinates (“scores”), which represent the sample distribution patterns and contributions (“loadings”), which indicate the influence of each variable on the observed distribution. Violin oxplots were built using the ‘ggplot2’ [34] (R software– version 4.3.3) package.

Pearson correlation coefficients between the specific water parameters (levels of turbidity and permanganate index, PI) and the concentration of individual PhACs were determined to highlight strong correlations (|corr| > 0.6). Statistical significance was performed using the ‘corrplot’ [35] (R software) package.

## 4. Conclusions

Turbidimetry and permanganate index, two measures of water quality, influence the presence, stability, or extraction efficiency of PhACs by the SPE procedure.

The SPE sorbent may physically become blocked by a high concentration of organic matter (>PI), reducing the levels of PhACs quantified by UPLC-MS/MS. However, higher PhAC concentrations were also observed in samples with higher TUR, which can be explained by the co-extraction of target compounds from particles bound to them during SPE.

Filtration is not an essential step in the analysis of PhACs by SPE-UPLC-MS/MS. The quantification of PhACs in filtered and unfiltered wastewater samples yields results that are not significantly different, nor do they exhibit a marked positive or negative trend. However, a tendency is observed in filtered samples where they show increased values for compounds with higher overall concentrations in the samples. Therefore, from a quantitative point of view, filtration is an option for the laboratory. However, results for filtered samples are higher for compounds with higher concentrations. As filtration also reduces the load of potential interferents, thus increasing the stability and lifetime of the chromatographic column, and minimizes the potential effects of ion suppression or enrichment, the best option is to use the filtration step.

## Figures and Tables

**Table 1:** Turbidity and permanganate index of unfiltered water samples, wastewater influent (WWI) and effluent (WWE) from seven WWTPs, and surface water (upstream and downstream).

Sample	Turbidity (NTU)						
	Tr	SM	PenM	S	VNB	E	SC
WWI	349	189	94.1	95.8	40.9	266	734
	255	278	91.4	150	395	363	500
WWE	150	102	46.6	39.9	39.1	1.36	2.94
	17.37	13.5	6.49	3.38	15.3	0.7	1.72
Upstream	3.86	99	62.8	91.9	---	---	---
	0	3.71	29.4	---	---	---	---
Downstream	8.12	33.7	79.7	36	---	---	---
	2.46	6.38	64.5	---	---	---	---
**Sample**	**Permanganate Index (PI, mg/L O_2_)**						
	**Tr**	**SM**	**PenM**	**S**	**VNB**	**E**	**SC**
WWI	100.1	188.5	233.8	219.9	62.5	107.5	527.3
	51.9	43.2	52.4	74.8	44.2	73.7	671
WWE	222.9	178.4	234.4	165	34.1	16.1	31.7
	19.0	10.5	10.2	14.4	10.3	16.9	15.5
Upstream	282.7	224.2	255.1	165.5	---	---	---
	10.5	6.1	12.5	---	---	---	---
Downstream	233.8	214.9	199.6	217	---	---	---
	9.8	16.7	31.9	---	---	---	---

**Table 2:** Characterization of WWTPs, number of samples collected, and identification of wastewater and surface water sampling points.

Profile	Beira Baixa Region			Beira Alta Region			
	E WWTP	SC WWTP	VNB WWTP	PenM WWTP	SM WWTP	S WWTP	Tr WWTP
Population equivalent	25,000	79,832	8000	3143	40,000	21,000	10,000
Treatment	LT-ASP	LT-ASP	LT-ASP	LAAP	LAAP	LAAP	LAAP
Wastewater sources							
Domestic sewage	X	X	X	X	X	X	X
Industrial sewage	X	X	X	X	X	X	X
Hospital sewage							
Number of samples							
Influents	2	2	2	2	2	2	2
Effluents	2	2	2	2	2	2	2
Upstream	---	---	---	2	2	1	2
Downstream	---	---	---	2	2	1	2
Total	4	4	4	8	8	6	**8**
Receiving waters	Santa Catarina stream	Bezelga stream	Borba stream	Penela stream	Diz river	Seia river	Noémi river

LT-ASP (long-term activate sludge process); LAAP (activated sludge under prolonged aeration).

## Data Availability

The data on PhACs in wastewater are private due to the identification of sampling points.

## Supplementary Materials

The following supporting information can be downloaded at: https://www.mdpi.com/article/10.3390/molecules30071609/s1. Table S1. Minimum (Min), maximum (Max), average (Avg), and median (Med) concentrations of PhACs. Per-centage of positive results (Pos (%)) and frequency (Freq) of detection in filtered and unfiltered influent wastewater samples from seven Portuguese WWTPs (n = 14). Table S2. Minimum (Min), maximum (Max), average (Avg), and median (Med) concentrations of PhACs. Per-centage of positive results (Pos (%)) and frequency (Freq) of detection in filtered and unfiltered effluent wastewater samples from seven Portuguese WWTPs (n = 14). Table S3. Minimum (Min), maximum (Max), mean (Avg) and median (Med) concentrations of PhACs. Percent-age of positive results (Pos (%)) and frequency (Freq) of detection in surface waters upstream of the 7 Portuguese WWTPs, filtered and unfiltered samples (n = 7). Table S4. Minimum (Min), maximum (Max), mean (Avg) and median (Med) concentrations of PhACs. Percentage of positive results (Pos (%)) and frequency (Freq) of detection in surface waters downstream of the 7 Portuguese WWTPs, filtered and unfiltered samples (n = 7).
