# Supplementary material for: Analysis of Pharmaceutical Active Compounds in Complex Water Samples: Sample Filtration as an Option
Source: Molecules. 2025 Apr 3;30(7):1609. doi: 10.3390/molecules30071609 (PMC11990622; doi:10.3390/molecules30071609)
Supplement: Supplementary file 1 [file molecules-30-01609-s001.zip › molecules-3536759-supplementary.pdf]

molecules

MDPI

Article

Analysis of pharmaceutical active compounds in complex water samples: sample filtration as an option

Sofia Silva [1], João Rodrigues [2], Vitor V Cardoso [3], Rui N Carneiro [4] and Cristina M M Almeida [5*]

[1] Empresa Portuguesa das Águas Livres, S.A. – EPAL, Direção de Laboratórios, Lisboa, Portugal; sofia.silva-e@adp.pt
[2] CICECO – Aveiro Institute of Materials, Department of Chemistry, University of Aveiro, Campus Universitário de Santiago, 3810-193 Aveiro, Portugal; joao.rodrigues@ua.pt
[3] Empresa Portuguesa das Águas Livres, S.A. – EPAL, Direção de Laboratórios, Lisboa, Portugal; vitor.cardoso@adp.pt
[4] Empresa Portuguesa das Águas Livres, S.A. – EPAL, Direção de Laboratórios, Lisboa, Portugal; rui.carneiro@adp.pt
[5] iMed.UL, Faculdade de Farmácia da Universidade de Lisboa, Lisboa, Portugal.
* Correspondence: calmeida@ff.ulisboa.pt

Supplementary Material

Table S1. Minimum (Min), maximum (Max), average (Avg), and median (Med) concentrations of PhACs. Percentage of positive results (Pos (%)) and frequency (Freq) of detection in filtered and unfiltered influent wastewater samples from seven Portuguese WWTPs (n= 14).

**Acidic chromatographic method**												
**Influent (µg/L)**												
	**Filtred Samples**						**Unfiltered samples**					
**PhAC**	**Min**	**Max**	**Avg**	**Med**	**Pos (%)**	**Freq**	**Min**	**Max**	**Avg**	**Med**	**Pos (%)**	**Freq**
ATN	0.041	2.200	0.600	0.363	100	2.0	0.045	1.70	0.520	0.431	100	2.0
APAP	23.0	193.9	77.0	61.9	100	2.0	24.6	149.5	65.5	47.9	100	2.0
SDZ	0.599	0.599	0.599	0.599	7	0.1	0.049	0.642	0.345	0.345	14	0.3
SPD	0.084	1.800	0.719	0.626	86	1.7	0.125	1.20	0.595	0.450	86	1.7
CAF	16.0	77.4	39.4	35.7	100	2.0	9.60	99.2	39.3	35.2	100	2.0
SMX	0.051	1.500	0.493	0.331	71	1.4	0.040	1.21	0.409	0.295	71	1.4
MTPL	0.040	0.195	0.094	0.087	43	0.9	0.041	0.142	0.075	0.067	43	0.9
PPNL	0.049	0.123	0.079	0.083	71	1.4	0.041	0.177	0.079	0.076	71	1.4
CTS	0.054	0.061	0.058	0.058	14	0.3	0.056	0.056	0.056	0.056	7	0.1
CBZ	0.157	1.190	0.415	0.313	100	2.0	0.141	1.20	0.464	0.356	93	1.9
CFA	---	---	---	---	0	0	---	---	---	---	0	0
NPX	0.643	38.5	9.442	4.45	100	2.0	0.688	37.9	8.64	3.85	100	2.0
GTD	0.046	0.046	0.046	0.046	7	0.1	---	---	---	---	0	0
TTE	0.030	0.034	0.032	0.032	7	0.3	0.035	0.084	0.059	0.059	14	0.3
**Basic chromatographic method**												
BZF	0.065	4.000	0.887	0.614	100	2.0	0.060	2.700	0.800	0.600	93	1.9
IBUP	1.900	30.5	11.857	8.80	100	2.0	1.800	28.200	9.664	6.500	100	2.0
DCF	0.058	5.20	2.042	1.850	100	2.0	0.644	4.100	1.931	1.775	100	2.0
E1	---	---	---	---	0	0	---	---	---	---	0	0
E3	0.362	0.538	0.450	0.450	14	0.3	0.472	0.784	0.636	0.654	21	0.4
E2	---	---	---	---	0	0	---	---	---	---	0	0
EE2	---	---	---	---	0	0	---	---	---	---	0	0
DES	---	---	---	---	0	0	---	---	---	---	0	0
ERT	0.087	0.111	0.099	0.099	14	0.3	---	---	---	---	0	0
FLX	---	---	---	---	0	0	0.070	0.124	0.088	0.071	21	0.4
CLR	0.076	0.706	0.309	0.232	71	1.4	0.077	0.784	0.263	0.152	71	1.4
AZM	0.092	2.300	0.651	0.507	93	1.9	0.073	4.700	0.725	0.331	100	2.0

--- (< Method Quantification Limit (MQL))

Table S2. Minimum (Min), maximum (Max), average (Avg), and median (Med) concentrations of PhACs. Percentage of positive results (Pos (%)) and frequency (Freq) of detection in filtered and unfiltered effluent wastewater samples from seven Portuguese WWTPs (n= 14).

	Acidic chromatographic method											
	Effluent (µg/L)											
	Filtred Samples						Unfiltered samples					
**PhAC**	**Min**	**Max**	**Avg**	**Med**	**Pos (%)**	**Freq**	**Min**	**Max**	**Avg**	**Med**	**Pos (%)**	**Freq**
ATN	0.061	0.220	0.124	0.112	64	1.3	0.039	0.353	0.139	0.039	71	1.4
APAP	0.050	21.9	4.315	0.120	50	1.0	0.064	23.6	4.41	0.064	57	1.1
SDZ	---	---	---	---	0	0	---	---	---	---	0	0
SPD	0.057	0.728	0.235	0.144	79	1.6	0.062	0.671	0.250	0.062	79	1.6
CAF	0.094	30.6	4.101	0.160	100	2.0	0.065	28.2	3.69	0.065	100	2.0
SMX	0.040	0.236	0.110	0.090	86	1.7	0.045	0.223	0.108	0.045	71	1.4
MTPL	0.047	0.185	0.094	0.051	21	0.4	0.045	0.192	0.080	0.045	36	0.7
PPNL	0.043	0.107	0.064	0.050	43	0.9	0.044	0.075	0.060	0.044	50	1.0
CTS	---	---	---	---	0	0	---	---	---	---	0	0
CBZ	0.048	0.811	0.444	0.447	100	2.0	0.233	0.938	0.571	0.233	93	1.9
CFA	---	---	---	---	0	0	---	---	---	---	0	0
NPX	0.040	9.800	1.362	0.204	100	2.0	0.049	9.50	1.37	0.049	100	2.0
GTD	---	---	---	---	0	0	---	---	---	---	0	0
TTE	0.040	0.065	0.052	0.052	14	0.3	0.034	0.071	0.053	0.034	14	0.3
	**Basic chromatographic method**											
BZF	0.069	3.20	0.981	0.257	57	1.1	0.059	4.200	1.12	0.059	57	1.1
IBUP	---	9.30	2.485	1.257	36	0.7	0.176	9.70	2.97	0.176	43	0.9
DCF	0.341	4.70	1.996	1.680	100	2.0	0.454	3.90	2.29	0.454	100	2.0
E1	---	---	---	---	0	0	---	---	---	---	0	0
E3	---	---	---	---	0	0	---	---	---	---	0	0
E2	---	---	---	---	0	0	---	---	---	---	0	0
EE2	---	---	---	---	0	0	---	---	---	---	0	0
DES	---	---	---	---	0	0	---	---	---	---	0	0
ERT	---	---	---	---	0	0	---	---	---	---	0	0
FLX	---	---	---	---	0	0	---	---	---	---	0	0
CLR	0.078	0.670	0.243	0.112	29	0.6	0.097	0.641	0.222	0.097	36	0.7
AZM	0.073	0.566	0.220	0.193	50	1.0	0.075	0.764	0.318	0.075	71	1.4

--- (< MQL)

Table S3. Minimum (Min), maximum (Max), mean (Avg) and median (Med) concentrations of PhACs. Percentage of positive results (Pos (%)) and frequency (Freq) of detection in surface waters upstream of the 7 Portuguese WWTPs, filtered and unfiltered samples (n= 7).

Acidic chromatographic method												
Upstream surface waters (µg/L)												
	Filtred Samples						Unfiltered samples					
**PhAC**	**Min**	**Max**	**Avg**	**Med**	**Pos (%)**	**Freq**	**Min**	**Max**	**Avg**	**Med**	**Pos (%)**	**Freq**
ATN	0.415	0.415	0.415	0.415	8	0.14	0.250	0.250	0.250	0.250	8	0.14
APAP	0.112	23.600	11.856	11.856	17	0.29	0.060	48.400	12.157	0.085	33	0.57
SDZ	---	---	---	---	0	0	---	---	---	---	0	0
SPD	---	---	---	---	0	0	---	---	---	---	0	0
CAF	0.087	19.000	2.913	0.126	58	1.00	0.065	37.700	5.734	0.093	58	1
SMX	0.060	0.060	0.060	0.060	8	0.14	---	---	---	---	0	0
MTPL	---	---	---	---	0	0	---	---	---	---	0	0
PPNL	0.060	0.060	0.060	0.060	8	0.14	0.099	0.099	0.099	0.099	8	0.14
CTS	---	---	---	---	0	0	---	---	---	---	0	0
CBZ	0.175	0.175	0.175	0.175	8	0.14	0.219	0.219	0.219	0.219	8	0.14
CFA	---	---	---	---	0	0	---	---	---	---	0	0
NPX	0.241	3.000	1.621	1.621	17	0.29	0.235	2.900	1.568	1.568	17	0.29
GTD	---	---	---	---	0	0	---	---	---	---	0	0
TTE	---	---	---	---	0	0	---	---	---	---	0	0
**Basic chromatographic method**												
BZF	0.091	0.133	0.112	0.112	17	0.29	0.095	0.133	0.114	0.114	17	0.29
IBUP	0.177	2.700	1.439	1.439	17	0.29	0.094	2.200	1.147	1.147	17	0.29
DCF	0.161	2.200	1.181	1.181	17	0.29	0.114	0.639	0.376	0.376	17	0.29
E1	---	---	---	---	0	0	---	---	---	---	0	0
E3	---	---	---	---	0	0	---	---	---	---	0	0
E2	---	---	---	---	0	0	---	---	---	---	0	0
EE2	---	---	---	---	0	0	---	---	---	---	0	0
DES	---	---	---	---	0	0	---	---	---	---	0	0
ERT	---	---	---	---	0	0	---	---	---	---	0	0
FLX	---	---	---	---	0	0	---	---	---	---	0	0
CLR	---	---	---	---	0	0	---	---	---	---	0	0
AZM	---	---	---	---	0	0	0.095	0.095	0.095	0.095	8	0.14

--- (< MQL)

Table S4. Minimum (Min), maximum (Max), mean (Avg) and median (Med) concentrations of PhACs. Percentage of positive results (Pos (%)) and frequency (Freq) of detection in surface waters downstream of the 7 Portuguese WWTPs, filtered and unfiltered samples (n= 7).

Acidic chromatographic method												
Downstream surface waters (µg/L)												
	Filtred Samples						Unfiltered samples					
**PhAC**	**Min**	**Max**	**Avg**	**Med**	**Pos (%)**	**Freq**	**Min**	**Max**	**Avg**	**Med**	**Pos (%)**	**Freq**
ATN	0.000	0.053	0.026	0.026	8	0.14	0.058	0.058	0.058	0.058	8	0.14
APAP	0.045	0.077	0.061	0.061	17	0.29	0.132	0.317	0.225	0.225	17	0.29
SDZ	---	---	---	---	0	0	---	---	---	---	0	0
SPD	---	---	---	---	0	0	---	---	---	---	0	0
CAF	0.075	3.400	0.787	0.124	50	0.86	0.079	4.300	0.819	0.115	50	0.86
SMX	---	---	---	---	0	0	---	---	---	---	0	0
MTPL	---	---	---	---	0	0	0.066	0.066	0.066	0.066	8	0.14
PPNL	---	---	---	---	0	0	---	---	---	---	0	0
CTS	---	---	---	---	0	0	---	---	---	---	0	0
CBZ	0.043	0.060	0.051	0.051	33	0.57	0.041	0.068	0.052	0.046	25	0.43
CFA	---	---	---	---	0	0	---	---	---	---	0	0
NPX	0.035	1.420	0.510	0.074	25	0.43	0.039	1.500	0.526	0.040	25	0.43
GTD	---	---	---	---	0	0	---	---	---	---	0	0
TTE	---	---	---	---	0	0	---	---	---	---	0	0
**Basic chromatographic method**												
BZF	0.366	0.219	0.234	0.366	25	0.43	0.062	0.298	0.180	0.180	17	0.29
IBUP	1.01	0.635	0.635	1.01	17	0.29	1.400	1.400	1.40	1.40	8	0.14
DCF	0.378	0.168	0.113	0.378	42	0.71	0.076	0.478	0.182	0.107	42	0.71
E1	---	---	---	---	0	0	---	---	---	---	0	0
E3	---	---	---	---	0	0	---	---	---	---	0	0
E2	---	---	---	---	0	0	---	---	---	---	0	0
EE2	---	---	---	---	0	0	---	---	---	---	0	0
DES	---	---	---	---	0	0	---	---	---	---	0	0
ERT	---	---	---	---	0	0	---	---	---	---	0	0
FLX	---	---	---	---	0	0	---	---	---	---	0	0
CLR	---	---	---	---	0	0	---	---	---	---	0	0
AZM	---	---	---	---	0	0	---	---	---	---	0	0

--- (< MQL)
